# Association of non-chronic low back pain with physical function, endurance, fatigability, and quality of life in middle- and older-aged adults: Findings from Baltimore Longitudinal Study of Aging

**DOI:** 10.1371/journal.pone.0277083

**Published:** 2022-11-10

**Authors:** Tatiana Rehder Gonçalves, Diana Barbosa Cunha, Mauro F. F. Mediano, Amal A. Wanigatunga, Eleanor M. Simonsick, Jennifer A. Schrack

**Affiliations:** 1 Institute of Social Medicine, State University of Rio de Janeiro, Rio de Janeiro, RJ, Brazil; 2 Department of Epidemiology, Johns Hopkins Bloomberg School of Public Health, Baltimore, MD, United States of America; 3 Evandro Chagas National Institute of Infectious Disease, Oswaldo Cruz Foundation, Rio de Janeiro, RJ, Brazil; 4 Center on Aging and Health, Johns Hopkins University, Baltimore, MD, United States of America; 5 National Institute on Aging, National Institutes of Health, Baltimore, MD, United States of America; Yamaguchi University: Yamaguchi Daigaku, JAPAN

## Abstract

Low back pain (LBP) is an important condition associated with high healthcare burden. However, the relationship of this condition with physical function (PF) and health-related quality of life (HRQoL) remains unclear. This is a cross-sectional study that aims to investigate the association between presence and intensity of non-chronic LBP with PF and HRQoL in middle-and older-aged adults. Participants answered questions about presence and intensity of LBP in the previous year, self-reported their PF and HRQoL (SF-12), and underwent objective measures of PF ([ExSPPB] including usual gait speed, narrow walk, chair stands, and standing balance), endurance performance ([EP] long-distance corridor walk) and fatigability. Generalized linear models and logistic regression models were performed. A total of 1500 participants (52.5% women, 70.3% white) aged 69.0 (SD 13.1) years were included. Of those, 642 (42.8%) reported LBP and the mean pain intensity was 4.1 (SD 2.2). After adjustments for potential confounders, presence of LBP was associated with lower self-reported PF (OR 1.73, 95% CI 1.27 to 2.37), lower physical component of HRQoL (β -0.03, 95% CI -0.04 to -0.02) and poorer chair stand performance (β -0.05, 95% CI -0.09 to -0.008). Higher intensity of LBP was associated with lower physical component of HRQoL (β -0.01, 95% CI -0.02 to -0.007), poorer ExSPPB performance (β -0.01, 95% CI -0.02 to -0.004), slower usual gait speed (β -0.01, 95% CI -0.02 to -0.004), lower total standing balance time (β -0.01, 95% CI -0.02 to -0.001) and higher fatigability (OR 1.13, 95% CI 1.01 to 1.25). The presence of non-chronic LBP was more consistently associated with lower self-reported PF, while higher intensity non-chronic LBP was associated with poorer objectively measured PF and fatigability. Collectively, this evidence suggests that although presence of pain may affect perception of function, greater pain intensity appears more strongly associated with unfavorable functional performance in mid-to-late life.

## Introduction

Low back pain (LBP) is a common condition affecting people of all ages, with an estimated lifetime prevalence of 70 to 85% [1]. The healthcare burden associated with LBP is high, not only due to direct costs (medical appointments, exams, medications, and hospitalizations) but also loss of work productivity [2, 3]. However, despite LBP’s clinical and economic significance, studies examining the relationship between the presence and intensity of LBP with reported and observed physical function and health related quality of life (HRQoL) have had inconsistent findings [4–12].

A cross-sectional comparative study of 320 older adults with LBP and healthy controls found that persons with LBP had more self-reported disability and lower objectively-measured physical function than their pain-free counterparts [4]. Similarly, a matched case-control study using objective measures of physical function demonstrated that participants with LBP with radiculopathy were energetically less efficient, had decreased endurance performance and slower gait speed compared to controls without pain [5]. However, a cross-sectional survey of 2,766 well-functioning community-dwelling older adults found a negative association between LBP and self-reported, but not objectively-measured physical function [6]. Moreover, although some authors have demonstrated an impact of LBP on endurance performance [7], a recent study did not confirm significant difference for endurance performance between individuals with and without non-specific LBP [8]. These previous studies primarily included patients with chronic LBP (>12 weeks), with the relationship between LBP and functional outcomes in patients with non-chronic LBP (<12 weeks) still underreported.

The inconsistent results observed in the literature may be attributed to the use of different tools to evaluate physical function (self-reported vs. objectively-measured instruments), that do not uniformly assess the same constructs [13], as definitions of physical function can range from perceptions of fundamental and complex functional abilities [6, 14] to direct functional evaluations including gait, balance, speed, reaction time, and endurance performance [6, 15]. In addition, objectively performance tests usually assess absolute levels of function which may be less sensitive in younger adults and higher functioning older adults, whereas self-reported usually measures relative function.

HRQoL, defined as a self-evaluation of well-being, considers the influence of a variety of health conditions and their impact on health status [16]. Previous researches suggests that people with LBP may present poor HRQoL [9] due to physical and psychological distress [17] and poor physical functioning [10], although others did not confirm these associations [11, 12]. Therefore, the impact of LBP on HRQoL is not well established in the literature.

Another important construct that has been associated with physical function but not yet explored in people with LBP is fatigability, a new concept that evaluates the severity of fatigue after performing a standardized task [18, 19]. Studies demonstrated that fatigability may act as an early biomarker of functional decline associated with reduced physical function and activity [20, 21]. Therefore, a better understanding of how LBP and fatigability are associated may improve our comprehension of the functional impacts of LBP, especially among higher functioning populations who may be more amenable to intervention efforts.

The present study aimed to investigate the association between the presence and intensity of non-chronic LBP with physical function, endurance performance, fatigability and HRQoL in middle- and older-aged adults. We hypothesized that both the presence and the intensity of non-chronic LBP would be negatively associated with functional parameters.

## Methods

This is a cross-sectional analysis including a subset of participants from Baltimore Longitudinal Study of Aging (BLSA) evaluated between August 2007 (when the fatigability test was initiated) and March 2018. Briefly, BLSA volunteers are community-dwelling adults submitted to comprehensive health and functional evaluations and free of major chronic conditions and cognitive and functional impairment at the time of enrollment [22]. The BLSA is a sample of convenience, and all testing takes place over the course of a 2.5-day visit. The study protocol was approved by the National Institutes of Health Intramural Institutional Review Board. Informed written consent was obtained from all participants. For the present study, we excluded participants aged <40 years and those that reported chronic pain (> 12 consecutive weeks) [23] according to the following question: “In the past year, what is the longest consecutive time period (in week) that you have had LBP?”. Participants with chronic LBP were exclude because they have specific characteristics regarding pain sensitization that may differentially impact physical function and HRQoL [24].

### Exposures

#### Presence and intensity of non-chronic LBP

The presence of LBP in the past year was assessed by an interviewer-administered question “In the past year, have you had any LBP?” with yes/no options. For those that reported LBP in the past year, intensity was assessed by the following question: “Please rate your usual back pain over the past year using a scale from 0 to 10, where 0 indicates no pain and 10 indicates extremely intense pain”. Intensity was analyzed as a continuous variable [25]. Participants that reported moderate to severe pain (rated >4) of any type during their pre-visit screen were encouraged to postpone their clinic visit to minimize potential interferences in the performance of physical functioning tests [26].

### Outcomes

#### Self-reported physical function

Self-reported physical function was assessed during a health interview to gauge ability to perform basic and instrumental activities of daily living. All 16 questions started with “Because of a health or physical problem, do you have any difficulty…”. The answers options were “yes”, “no”, “don’t know/don’t do” and “refused”. Participants answering “don’t know / don’t do” or “refused” were excluded from the analyses [27]. Participants were classified as having low self-reported physical function if they answered “yes” to at least one of the questions (dichotomous variable).

#### HRQoL

HRQoL was assessed by the 12 Item Short Form Medical Outcomes Survey (SF-12) [28], a short questionnaire derived from the Short Form-36 Health Survey (SF-36) [29] including 12 questions about 8 dimensions of health and physical functioning (limitations due to physical health problems, bodily pain, general health, vitality, social functioning, role limitations due to emotional problems, and mental health) that provide scales for physical and mental health components. Scores of physical component summary (PCS-12) and mental component summary (MCS-12) continuously range from 0 (as the lowest) to 100 (as the highest) [28].

#### Objectively-measured physical function

Objectively-measured physical function was assessed using the Expanded Short Physical Performance Battery (ExSPPB), which comprises a battery of four performance tests as follows: 1) *Usual Gait Speed*: participants were invited to walk at their “usual walking pace” over a 6-meter course and the faster of two trials was used for analysis; 2) *Narrow Walk*: the ability and time to walk a course measuring 6 meters long and 20 centimeters wide. Stepping on or outside of the tape two or more times constituted a failure. Up to three attempts were allowed to obtain a valid time; 3) *Chair Stands*: time to stand-up and sit down on an armless chair 5 times; 4) *Standing Balance*: ability to hold three progressively more challenging standing balance poses (semi-tandem, full tandem and single leg) for up to 30 seconds each. The performance of each test generated a ratio score that was analyzed as a separate outcome. The ratio score was calculated using the maximal performance as the denominator and individual performance as the numerator. The overall ExSPPB score was calculated as the sum of the ratio scores obtained in each test for a continuous scale ranging from 0 (the lowest) to 4 (the highest) [20, 27, 30].

#### Endurance performance

Endurance performance was evaluated using the long-distance corridor walk (LDCW). The LDCW is a self-paced endurance walking test and validated measure of cardiorespiratory fitness [30, 31]. The test was performed on a 20-meter uncarpeted corridor marked by cones at both ends. Participants were instructed to walk as quickly as possible over the full 10 laps. Standardized encouragement was given with each lap along with the number of laps remaining. Ability to complete the LDCW test in <5 min was examined as a dichotomous outcome [30, 32]. Endurance performance was measured as the continuous time to walk 400 meters.

#### Fatigability

Perceived fatigability was assessed immediately after a 5-min, 1.5 mph (0.67 m/s) standardized treadmill walk by asking participants to rate their perceived exertion using the Borg perceived exertion scale. The scale ranges from 6 to 20, where 6 refers to “no exertion at all”, 9 to “very light”, 11 to “light”, 12 to “somewhat hard” and 20 to “maximal exertion” [18, 33]. Perceived fatigability was explored as a continuous and dichotomous variable (high fatigability ≥10) in the analyses [18, 20].

### Covariates

Adjustments for potential confounders were performed to determine the independent association between non-chronic LBP and outcomes, including sociodemographic (age and race), anthropometric (body mass index), lifestyle (history of smoking, hours of sleep and physical activity) and clinical variables (comorbidities and painful regions in the body) [2, 34–37]. Standardized procedures were used to assess height and weight using a stadiometer and a calibrated scale, to calculate body mass index (BMI = kg/m^2^). The following variables were extracted from a health history interview that was conducted by a nurse practitioner during the clinic visits. Age was considered as continuous variable. Self-reported race was categorized as white or non-white. Height and weight were used to calculate body mass index (BMI = kg/m^2^). Self-reported smoking was categorized as never or current/past smoker. The presence of depression was self-reported and treated as a dichotomous variable (yes/no). Hours of sleep was dichotomized into ≥7h or <7h per night [38]. Number of comorbidities consisted of the sum of self-reported cardiovascular disease, pulmonary disease, cerebral vascular disease, peripheral neuropathy, hypertension, diabetes, cancer, and arthritis, and categorized as none, one/two or more.

Number of painful sites was created considering any pain or discomfort on different body sites including head, neck, shoulder, wrists, hands, legs, hip, knee, feet, toes and ankles. This sum was categorized as none, one, two, or three/more painful sites. Physical activity (PA) was assessed by a self-reported questionnaire that included 17 items regarding daily activities and programmed exercise. Metabolic equivalent (METs) was generated based on the coding described by Ainsworth et al. that combined the frequency and duration spent in each activity during the previous two weeks to generate the total volume of PA, expressed as a continuous variable (MET*min/week) [39].

### Statistical analyses

Descriptive statistics for continuous variables comprised means and standard deviations and categorical variables comprised frequency and percentage. Associations between exposure variables (presence and intensity of non-chronic LBP) were analyzed separately as different exposures with the outcomes (self-reported physical function and HRQoL and objectively-measured physical function). Models were fitted crude and adjusted by sex, age, race, BMI, smoking, self-reported depression, hours of sleep per night, comorbidity count, number of painful sites and PA. Generalized linear models with log link and gamma distribution to account for potentially skewed distributions were utilized for the continuous outcomes (PCS-12, MCS-12, ExSPPB, usual gait speed, narrow walk time, chair stands, standing balance time, time to complete 400m and fatigability). The logged beta values from the variables that were statistically significant were exponentiated to facilitate the interpretation of the results. For dichotomous outcomes, logistic regression models were used for self-reported physical function, high fatigability and the ability to complete the 400m in < 5 min. All analyses were performed using Stata 13.0. Statistical significance was set at a 2-tailed p-value of <0.05 for all analyses.

## Results

**Fig 1:**
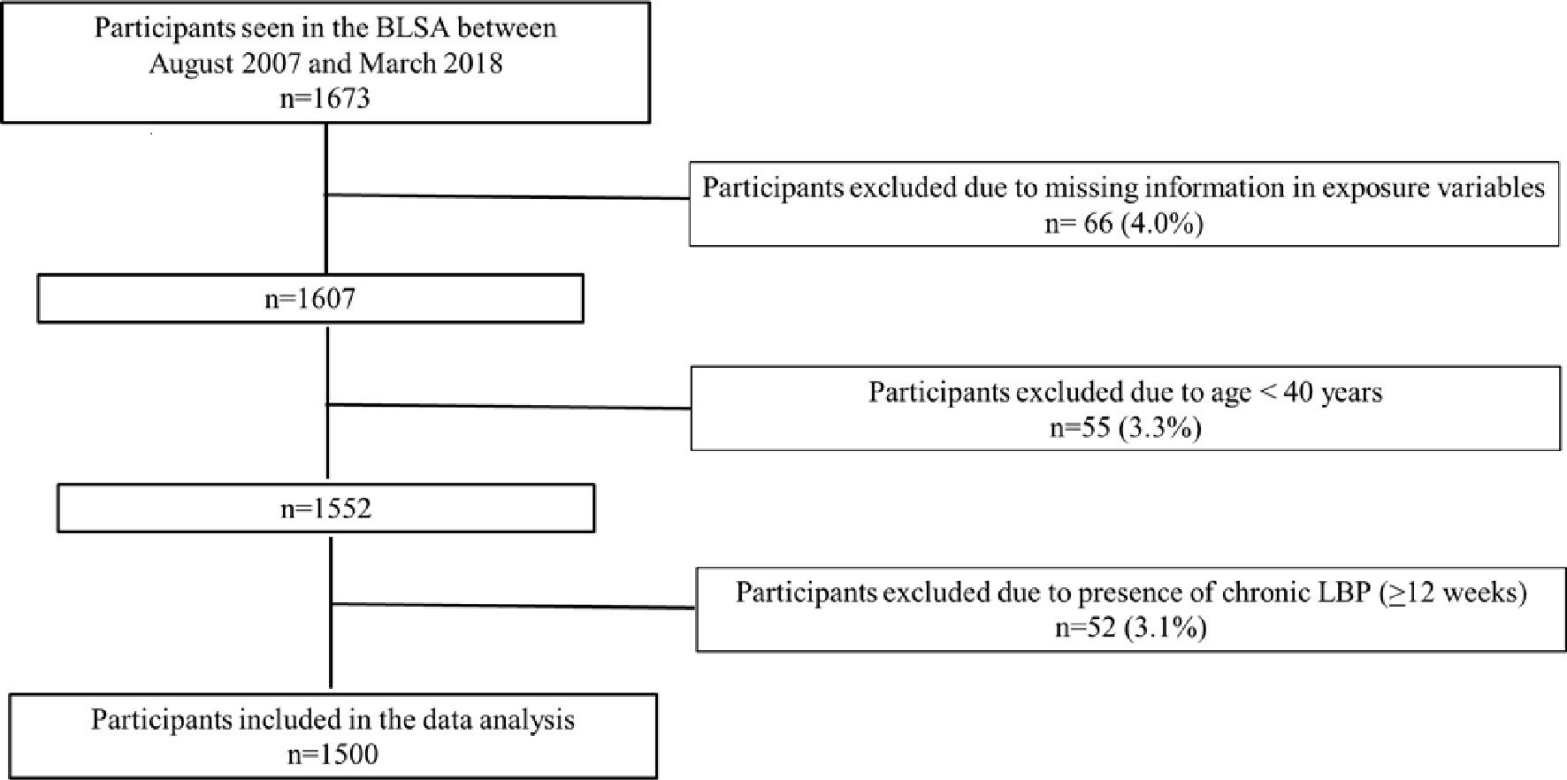
Participants flow chart.

Of the 1,673 participants of the BLSA between August 2007 and March 2018, 66 were excluded due to missing information in exposure variables, 55 aged < 40 years, and 52 reported chronic LBP (≥12 weeks) (Fig 1). Table 1 describes the characteristics of the 1,500 participants included in the study population, overall and stratified by non-chronic LBP status. The mean age was 69.0 (±13.1) years, 52.5% were women and 70.3% were white. Participants reporting non-chronic LBP in the previous year (42.8%) were younger (67.4 ±12.4 vs 70.1 ±13.5 years), have a higher BMI (27.8 ±5.1 vs 26.9 ±4.8 kg/m^2^), were more likely to self-report depression (21.2% vs 13.3%), and have a higher percentage of ≥3 painful sites (27.8% vs 10.1%) than participants without non-chronic LBP. The mean pain intensity among those reporting non-chronic LBP in the previous year was 4.1 (±2.2).

**Table 1:** Characteristics of participants according to non-chronic low back pain (NCLBP) status.

	Overall		No NCLBP		NCLBP		*p-*value
	(n = 1500)		(n = 858; 57.2%)		(n = 642; 42.8%)		
	Mean / n	SD / %	Mean / n	SD / %	Mean / n	SD / %	
Age (Years)	69.0	13.1	70.1	13.5	67.4	12.4	**<0.001**
Male sex	713	47.5%	415	48.4%	298	46.4%	0.45
White	1054	70.3%	598	69.7%	456	71.0%	0.58
Education level (Years) (n = 1499)	17.4	2.7	17.5	2.7	17.3	2.6	0.07
Body mass index (Kg/m^2^) (n = 1443)	27.2	5.0	26.9	4.8	27.8	5.1	**<0.001**
Ever smoked (n = 1490)	571	38.3%	315	36.9%	256	40.2%	0.20
Sleep hours > 7 (n = 1282)	511	39.9%	300	40.9%	211	38.5%	0.39
Depression (n = 1495)	249	16.7%	113	13.3%	136	21.2%	**<0.001**
Number of comorbidities							
0	508	33.9%	303	35.3%	205	31.9%	0.18
1	458	30.5%	266	31.0%	192	29.9%	
≥ 2	534	35.6%	289	33.7%	245	38.2%	
Physical activity (METs/min/week^-1^) (n = 1499)	107.9	92.7	106.1	103.0	110.3	76.7	0.38
Number of painful sites in the body (n = 1279)							
0	444	34.7%	319	43.4%	125	23.0%	**<0.001**
1	358	28.0%	205	27.9%	153	28.1%	
2	252	19.7%	137	18.6%	115	21.1%	
≥ 3	225	17.6%	74	10.1%	151	27.8%	
NCLBP intensity (n = 642)	-	-	-	-	4.1	2.2	-
Low Self-Reported Physical Function (n = 1278)	390	30.5%	176	24.3%	214	38.7%	**<0.001**
Health Related Quality of Life (SF-12) (n = 1438)							
*Physical Component Summary (PCS-12)*	52.2	7.4	53.3	6.5	50.7	8.2	**<0.001**
*Mental Component Summary (MCS-12)*	54.8	6.4	55.3	6.0	54.1	6.9	**<0.001**
Expanded Short Physical Performance Battery (ExSPPB) (n = 1280)	2.9	0.7	2.9	0.7	2.9	0.7	0.98
*Usual Gait Speed* (n = 1418)	1.1	0.3	1.1	0.3	1.1	0.3	0.77
*Narrow Walk Time (n = 1289)*	4.8	2.2	4.8	2.2	4.8	2.2	0.84
*Chair Stands (n = 1416)*	0.5	0.3	0.5	0.3	0.5	0.2	**0.05**
*Total Time Standing Balance (n = 1423)*	76.5	21.9	75.0	23.2	78.5	19.9	**0.003**
Long Distance Corridor Walk (LDCW)							
*LDCW test in < 5 min (n = 1268)*	1205	95%	691	95.1%	514	95.1%	0.98
*Time to Complete (n = 1205)*	268.4	55.3	266.9	52.8	270.5	58.5	0.28
Fatigability (n = 1220)							
*Continous Fatigability*	8.5	2.4	8.5	2.3	8.6	2.4	0.75
*High Fatigability*	308	25.3%	177	25.3%	131	25.2%	1.00

Table 2 shows the association between the presence and the intensity of non-chronic LBP with study outcomes. After adjustments for potential confounders, the presence of non-chronic LBP was associated with lower self-reported physical function (OR 1.73, 95% CI 1.27 to 2.37) and lower PCS-12 (β -0.03, 95% CI -0.04 to -0.02). For objective measures, presence of non-chronic LBP was associated only with lower chair stand performance (β -0.05, 95% CI -0.09 to -0.008). The exponentiated beta coefficient demonstrated that presence of non-chronic LBP was associated with a 3% reduction in the PCS-12 score and with a 5% reduction on the time to perform 5 chair stands.

**Table 2:** Association between presence and intensity of non-chronic LBP(NCLBP) with physical function, health related quality of life, endurance walking, and fatigability.

	Presence of NCLBP (yes / no)		NCLBP intensity	
	(n = 1500)		(n = 642; mean = 4.08, ±2.24)	
	Crude	Adjusted*	Crude	Adjusted*
**Low Self-Reported Physical Function—OR (95% CI)**	1.99 (1.57 to 2.54)	1.73 (1.27 to 2.37)	1.08 (1.00 to 1.17)	1.00(0.90 to 1.10)
	n = 1305	n = 1148	n = 564	n = 491
**Health Related Quality of Life (SF-12)**				
*Physical Component Summary (PCS-12)—β (95% CI)*	-0.05 (-0.07 to -0.04)	-0.03 (-0.04 to -0.02)	-0.02 (-0.02 to -0.01)	-0.01 (-0.02 to -0.007)
	n = 1438	n = 1238	n = 623	n = 528
*Mental Component Summary (MCS-12)—β (95% CI)*	-0.02 (-0.03 to -0.009)	-0.001 (-0.01 to +0.01)	-0.001 (-0.006 to +0.003)	-0.0006 (-0.004 to +0.005)
	n = 1438	n = 1238	n = 623	n = 528
**Expanded Short Physical Performance Battery (ExSPPB)**–**β (95% CI)**	0.001(-0.03 to +0.03)	-0.02 (-0.04 to +0.008)	-0.02 (-0.03 to -0.009)	-0.01 (-0.02 to -0.004)
	n = 1280	n = 1233	n = 546	n = 530
*Usual Gait Speed—β (95% CI)*	0.004 (-0.02 to +0.03)	-0.02 (-0.04 to +0.007)	-0.02 (-0.03 to -0.009)	-0.01(-0.02 to -0.004)
	n = 1418	n = 1239	n = 612	n = 531
*Narrow Walk Time—β (95% CI)*	0.005 (-0.05 to +0.06)	-0.006 (-0.06 to +0.04)	-0.02 (-0.04 to -0.0004)	-0.02 (-0.03 to +0.001)
	n = 1289	n = 1239	n = 548	n = 531
*Chair Stands—β (95% CI)*	-0.05 (-0.11 to -0.002)	-0.05 (-0.09 to -0.008)	-0.02 (-0.04 to -0.006)	-0.01 (-0.03 to +0.003)
	n = 1416	n = 1238	n = 612	n = 532
*Total Time Standing Balance—β (95% CI)*	0.05 (+0.02 to +0.08)	0.02 (-0.01 to +0.04)	-0.01 (-0.02 to -0.002)	-0.01 (-0.02 to -0.001)
	n = 1423	n = 1241	n = 614	n = 532
**Long Distance Corridor Walk (LDCW)**				
*LDCW test in < 5 min–OR (95% CI)*	1.00(0.72 to 1.41)	1.09 (0,70 to 1.70)	0.88 (0.79 to 0.98)	0.92 (0.79 to 1.07)
	n = 1268	n = 1241	n = 541	n = 531
*Time to Complete—β (95% CI)*	0.01(-0.01 to +0.04)	0.01(-0.06 to +0.03)	0.01(+0.004 to +0.02)	0.006 (-0.0002 to +0.01)
	n = 1205	n = 1180	n = 514	n = 505
**Fatigability**				
*Continous Fatigability—β (95% CI)*	0.005 (-0.26 to +0.04)	0.004 (-0.03 to +0.03)	0.01(-0.0006 to +0.21)	0.007 (-0.003 to +0.02)
	n = 1220	n = 1197	n = 519	n = 511
*High Fatigability—OR (95% CI)*	1.00(0.77 to 1.30)	0.99 (0.73 to 1.35)	1.12 (1.03 to 1.22)	1.13 (1.01 to 1.25)
	n = 1220	n = 1197	n = 519	n = 511

* Model adjusted by sex, age, race, body mass index (kg/m^2^), smoking history, self-reported depression, hours of sleep per night, number of comorbidities, number of painful sites and physical activity (MET*min*week)

For those that reported non-chronic LBP (n = 642), pain intensity was associated with lower PCS-12 (β -0.01, 95% CI -0.02 to -0.007) in adjusted models. Additionally, non-chronic LBP intensity was associated with a lower ExSPPB score (β -0.01, 95% CI -0.02 to -0.004), slower usual gait speed (β -0.01, 95% CI -0.02 to -0.004), lower total time standing balance (β -0.01, 95% CI -0.02 to -0.001) and higher rate of high fatigability (OR 1.13, 95% CI 1.01 to 1.25). The exponentiated beta coefficient demonstrated 1% reductions of PCS-12, ExSPPB, usual gait speed and total time standing balance scores for every increase in one unit of pain scale. No significant associations were observed for both presence and intensity of non-chronic LBP for the other study outcomes.

We also explored for effect modification by age categories (< 60 and ≥ 60 years), and no significant interaction was observed (S1 Table).

## Discussion

In the present study, the presence of non-chronic LBP was negatively associated with self-reported physical function, the physical component of HRQoL and objectively-measured repeated chair stands performance. Moreover, non-chronic LBP intensity was negatively associated with the physical component of HRQoL, and several objective measures of function including the ExSPPB, usual gait speed, total time standing balance and high fatigability. Collectively these results suggest that the presence of non-chronic LBP demonstrates an association with perceived functional abilities whilst greater pain intensity shows a stronger association with objectively-measured abilities.

There are some explanations for the presence of non-chronic LBP was more strongly associated with self-reported than objectively-measured tests. As suggested by Reuben et al. [13], self-reported and objectively-measured instruments evaluate different dimensions of physical function and do not necessarily measure the same constructs. Moreover, LBP is a multifactorial condition and its functional impact could be influenced by many different factors, including psychosocial and motivational aspects [40–43]. Self-report measurements evaluate activities that people usually perform in their daily, while objectively-measured instruments capture how well people can do a task during a single evaluation. Therefore, it is possible that people ignore or minimize their LBP during a performance-based assessment, which may not happen during the activities of daily living that are captured with a self-report measurement [44]. Moreover, individuals with LBP may be more reluctant to perform functional activities due to kinesiophobia (fear of movement), regardless of their real ability to perform them [45]. Also, self-reporting measurements are subjected to reporting bias, especially among those with pain, which may overestimate the impact of LBP on the performance of functional activities [46]. Another possible explanation is that the objectively-measured tests performed in the present study mostly evaluate lower extremity activities, not including specific movements of the spine required to perform several activities of daily living [6]. In this setting, the chair stand test maybe more demanding on trunk movements than other functional tests and this could explain why chair stands was the only objectively measure in the ExSPPB associated with non-chronic LBP, regardless of pain intensity [47, 48].

In a previous study from the BLSA, Simonsick et al. [26] observed that time to walk 400m, the measure termed endurance performance in this study, was slower with greater lumbopelvic pain severity experienced in the prior year. Our study demonstrated that other objectively-measured physical function components were also associated with non-chronic LBP intensity (ExSPPB, usual gait speed, total standing balance, and high fatigability), suggesting that more intense pain is associated with poorer performance of the activities that do not directly engage trunk movements. These findings are in accordance with a previous study that included 52 patients with LBP showing a significant inverse correlation between pain intensity with repeated squat and isometric lifting tests [49]. Similarly, pain intensity was significantly correlated with decreased physical function in a population with bilateral knee osteoarthritis, accounting to 18% of the overall variance in physical function, reinforcing the idea that greater pain intensities can be more debilitating, not only in individuals with LBP but also among those with other musculoskeletal pain [50].

The present study did not find any significant association between the presence and intensity of non-chronic LBP with endurance performance. Despite some previous studies have demonstrated that increased endurance performance is associated with lower risk of LBP, especially endurance in the back muscles, other studies did not confirm this association [51–53]. The lack of association observed in our study can likely be attributed to the characteristics of BLSA population, that is generally healthier and fitter than the general population, leading to a potential underestimation of the impact of non-chronic LBP on overall (not local back muscles) endurance performance [22]. Moreover, there was no association between the presence of non-chronic LBP and fatigability, although a negative association of high fatigability with non-chronic LBP intensity was observed. Conversely, some other studies have found a negative association between LBP and local muscle fatigability (e.g., diaphragm and lumbar muscles) but no one has evaluated the association between LBP and whole-body fatigability [54–56]. The lack of association between the presence of LBP and fatigability may be explained by the very low intensity of the fatigability test and the well-functioning characteristics of the BLSA population, in which greater pain intensities would be necessary to generate fatigue.

Our study found an inverse association for both presence and intensity of non-chronic LBP with the physical but not the mental component of HRQoL. These associations were examined in some other studies that also demonstrated a negative impact on the physical component [11, 57–59]. The association of LBP on physical component of HRQoL may be explained by the lower confidence of LBP individuals to perform the daily activities, as also demonstrated by the lower self-reported physical function. Since LBP is a multifactorial condition, it was expected that both physical and mental components would be negatively associated with LBP. The unexpected lack of association with mental HRQoL may also reflect the generally good health status of our study population.

Limitations of the present study include the cross-sectional study design that precludes temporal associations between non-chronic LBP (exposure) with physical function and HRQoL (outcomes). Therefore, it is not possible to determine a causal relationship due to the possibility of a bidirectional associations. Moreover, the objectively-measured tests performed in the present study mostly included movements from lower limbs, that could have limited our ability to detect other types of functional limitations due to non-chronic LBP. Also, the association between LBP intensity with functional outcomes and HRQoL may have been underestimated since participants reporting moderate to severe pain were encouraged to postpone their clinic visit. Finally, the question about the presence and intensity of LBP referred to any episode of LBP in the previous 12 months, representing a wide time period that increases the risk for misclassification. Strengths include the high-quality data provided by the BLSA and the inclusion of both self-reported and objectively-measured variables besides an overall measure of fatigability.

To conclude, the presence of non-chronic LBP was primarily associated with self-reported physical function and the physical domain of HRQoL. On the other hand, intensity of non-chronic LBP was negatively associated with the physical domain of HRQoL and with objectively-measured physical function, suggesting that pain intensity is an important dimension in evaluating potential threats to physical function. Longitudinal studies should be conducted to explore the impact of LBP on self-reported perception of functional ability and objective evaluation of capacity to perform functional activities, facilitating the development of effective approaches for preventing and managing LBP.

## Supporting information

S1 Table Association between presence of LBP and LBP intensity with physical function, health related quality of life, endurance walking, and fatigability stratified by age categories (<60 and ≥60 years old). (DOCX)
