# Supplementary material for: Association of non-chronic low back pain with physical function, endurance, fatigability, and quality of life in middle- and older-aged adults: Findings from Baltimore Longitudinal Study of Aging
Source: PLoS One. 2022 Nov 10;17(11):e0277083. doi: 10.1371/journal.pone.0277083 (PMC9648728; doi:10.1371/journal.pone.0277083)
Supplement: S1 Table — (DOCX) [file pone.0277083.s001.docx]

**Supplementary Table** – Association between presence of LBP and LBP intensity with physical function, health related quality of life, endurance walking, and fatigability stratified by age categories (<60 and >60 years old):

|  | **Presence of LBP** (yes / no)  (n=1500) | | | **LBP intensity**  (n=642; mean=4.08, +2.24) | | |
| --- | --- | --- | --- | --- | --- | --- |
|  | **<60 years**  (n=354) | **>60 years**  (n=1146) | **p-value for interaction** | **<60 years**  (n=162;  mean=4.12, +2.33) | **>60 years**  (n=480;  mean=4.07, +2.21) | **p-value for interaction** |
| **Low Self-Reported Physical Function –**  **OR (95% CI)** | 1.52  (0.68 to 3.40)  n=305 | 1.73  (1.23 to 2.44)  n=843 | 0.88 | 1.11  (0.85 to 1.44)  n=136 | 0.96  (0.86 to 1.08)  n=355 | 0.20 |
| **Health Related Quality of Life (SF-12)** |  | | | | | |
| *Physical Component Summary*  *(PCS-12) –*  *β (95% CI)* | -0.02  (-0.05 to +0.004)  n=308 | -0.03  (-0.05 to -0.02)  n=930 | 0.25 | -0.01  (-0.02 to -0.001)  n=137 | -0.01  (-0.02 to -0.004)  n=391 | 1.00 |
| *Mental Component Summary*  *(MCS-12) –*  *β (95% CI)* | -0.02  (-0.05 to +0.01)  n=308 | 0.002  (-0.01 to +0.02)  n=930 | 0.12 | -0.01  (-0.02 to -0.0003)  n=137 | -0.004  (-0.001 to +0.01)  n=391 | 0.07 |
| **Expanded Short Physical Performance Battery (ExSPPB)** **–**  **β (95% CI)** | -0.01  (-0.03 to +0.01)  n=307 | -0.02  (-0.05 to +0.01)  n=926 | 0.31 | -0.001  (-0.01 to +0.004)  n=137 | -0.02  (-0.03 to -0.004)  n=393 | 0.06 |
| *Usual Gait Speed - β (95% CI)* | -0.04  (-0.08 to -0.01)  n=309 | -0.01  (-0.04 to +0.02)  n=930 | 0.42 | -0.01  (-0.02 to +0.01)  n=137 | -0.01  (-0.02 to -0.003)  n=394 | 0.24 |
| *Narrow Walk Time –*  *β (95% CI)* | 0.05  (-0.01 to +0.12)  n=311 | -0.03  (-0.09 to +0.04)  n=929 | 0.07 | -0.01  (-0.03 to +0.02)  n=138 | -0.02  (-0.04 to +0.01)  n=393 | 0.48 |
| *Chair Stands –*  *β (95% CI)* | -0.03  (-0.10 to +0.05)  n=310 | -0.06  (-0.11 to -0.01)  n=928 | 0.56 | 0.01  (-0.02 to +0.03)  n=138 | -0.02  (-0.04 to -0.001)  n=394 | 0.07 |
| *Total Time Standing Balance - β (95% CI)* | 0.007  (-0.002 to +0.02)  n=310 | 0.01  (-0.03 to +0.05)  n=931 | 0.73 | -0.001  (-0.003 to +0.001)  n=138 | -0.01  (-0.02 to +0.001)  n=394 | 0.28 |
| **Long Distance Corridor Walk (LDCW)** |  | | | | | |
| *Time to Complete - β (95% CI)* | 0.01  (-0.01 to +0.04)  n=306 | 0.02  (-0.01 to +0.04)  n=874 | 0.67 | 0.01  (0.001 to 0.02)  n=136 | 0.003  (-0.004 to +0.01)  n=369 | 0.70 |
| **Fatigability** |  | | | | | |
| *Continous Fatigability - β (95% CI)* | 0.01  (-0.04 to +0.06)  n=301 | 0.01  (-0.03 to +0.04)  n=896 | 0.77 | 0.01  (-0.002 to +0.03)  n=135 | 0.004  (-0.01 to +0.02)  n=376 | 0.31 |
| *High Fatigability - OR (95% CI)* | 0.84  (0.34 to 2.07)  n=301 | 1.06  (0.76 to 1.48)  n=896 | 0.99 | 1,50  (1.04 to 2.16)  n=110 | 1.11  (0.99 to +1.25)  n=376 | 0.20 |

^*^ Model adjusted by sex, age, race, body mass index (kg/m^2^), smoking history, self-reported depression, hours of sleep per night, number of comorbidities, number of painful sites and physical activity (MET*min*week)
